# *In Vitro* Synergistic Effect of *Psidium guineense* (Swartz) in Combination with Antimicrobial Agents against Methicillin-Resistant *Staphylococcus aureus* Strains

**DOI:** 10.1100/2012/158237

**Published:** 2012-04-24

**Authors:** Tiago Gomes Fernandes, Amanda Rafaela Carneiro de Mesquita, Karina Perrelli Randau, Adelisa Alves Franchitti, Eulália Azevedo Ximenes

**Affiliations:** ^1^Laboratório de Fisiologia e Bioquímica de Microorganismos, Departamento de Antibióticos, Centro de Ciências Biológicas, Universidade Federal de Pernambuco, 50670-901 Recife, PE, Brazil; ^2^Laboratório de Farmacognosia, Departamento de Farmácia Universidade Federal de Pernambuco, Centro de Ciências da Saúde, 50670-901 Recife, PE, Brazil; ^3^Department of Biochemistry, Kansas State University, 141 Chalmers Hall, Manhattan, KS 66506, USA

## Abstract

The aim of this study was to evaluate the antimicrobial activity of aqueous extract of *Psidium guineense* Swartz (Araçá-do-campo) and five antimicrobials (ampicillin, amoxicillin/clavulanic acid, cefoxitin, ciprofloxacin, and meropenem) against twelve strains of *Staphylococcus aureus* with a resistant phenotype previously determined by the disk diffusion method. Four *S. aureus* strains showed resistance to all antimicrobial agents tested and were selected for the study of the interaction between aqueous extract of *P. guineense* and antimicrobial agents, by the checkerboard method. The criteria used to evaluate the synergistic activity were defined by the fractional inhibitory concentration index (FICI). All *S. aureus* strains were susceptible to *P. guineense* as determined by the microdilution method. The combination of the *P. guineense* extract with the antimicrobial agents resulted in an eight-fold reduction in the MIC of these agents, which showed a FICI ranging from 0.125 to 0.5, suggesting a synergistic interaction against methicillin-resistant *Staphylococcus aureus* (MRSA) strains. The combination of the aqueous extract of *P. guineense* with cefoxitin showed the lowest FICI values. This study demonstrated that the aqueous extract of *P. guineense* combined with beta lactamics antimicrobials, fluoroquinolones, and carbapenems, acts synergistically by inhibiting MRSA strains.

## 1. Introduction

The widespread emergence of bacteria resistance to a large number of antimicrobial agents poses major health problems because of difficulties in treatment [1]. *Staphylococcus aureus* is an important pathogen both in community acquired and nosocomial infections and a common etiological agent of infections of many different tissues and organs (e.g., furuncle, carbuncle, abscess, myocarditis, endocarditis, pneumonia, meningitis, bacterial arthritis, and osteomyelitis) [2]. The multidrug resistant *Staphylococcus aureus* strains now pose serious problems to hospitalized patients and their care providers. The organism has successfully evolved numerous strategies for resisting, the action to practically all antimicrobial agents [3]. Thus, it is extremely important to find new antimicrobial agents or new ways for the treatment of infectious diseases caused by multidrug resistant microorganisms [4].

The screening of plant extracts and phytochemicals for antimicrobial activity has shown that higher plants, especially their secondary metabolites, are a source to provide structurally diverse bioactive compounds with different pharmacological activities, including antimicrobials [5].

According to Hemaiswarya et al. [6], plants-derived antimicrobials are less potent, which enhances the need to adopt a synergistic interaction between its bioactive compounds to combat infections. Based on this knowledge, several studies have been published using crude plant extracts or phytochemicals combined with antimicrobial agents. These combinations can enhance the efficacy of the antimicrobial agents and are an alternative to treat infections caused by multidrug resistant microorganisms, which do not have any effective therapy available [4, 7, 8].

*Psidium guineense* Swartz (Myrtaceae), popularly known as wild guava, is a shrub native and widely dispersed throughout Tropical America. In Brazil, it mainly occurs across the coastline [9, 10].

Few studies have been focused on the phytochemical profile and pharmacological activity of this plant; however, it is widely used in folk medicine in regions of South America to treat infections of the gastrointestinal and genitourinary tract [11]. These authors associated the presence of mainly flavonoids and tannins in the fruits and leaves of *P. guineense* to be effective against *Streptococcus mutans* strains.

The aim of the present study was to evaluate the antimicrobial activity of the aqueous extract of *P. guineense* and to determine the synergistic potential of the combination of this extract with five antimicrobial agents against MRSA strains.

## 2. Materials and Methods

### 2.1. Plant Material and Extract Preparation

The plant materials used in this study consisted of *Psidium guineense* Swartz (leaves) that were collected in November/2009 from Moreno city, Pernambuco, Brazil, (08°07′07′′S-35°05′32′′W). This plant was identified by the biologist Olivia Cano, and the voucher specimen was deposited in the Herbarium Dárdano de Andrade Lima, Instituto Agronômico de Pernambuco and indexed under registration 83.564.

The leaves were air-dried for two weeks and then grinded into fine powder using an electric dry mill. A total of 50 g of the powder was soaked in 1000 mL of boiled distilled water and extracted exhaustively at room temperature for 48 hours. The extracts were filtered through Whatman No. 2 filter paper. The aqueous extract was lyophilized to obtain a dry powder extract.

### 2.2. Phytochemical Screening

The preliminary phytochemical screening was carried out by various secondary metabolites present on aqueous extract of *P. guineense *using standard procedures [12–14]. The chromatographic analysis was made by TLC on Si gel (MERCK, Germany, 105553) developed by different solvent systems: (EtOAc-HCOOH–AcOH-H_2_O (100 : 11 : 11 : 26, v/v)) to investigate the presence of saponins, flavonoids, tannins, phenylpropanoids, alkaloids, condensed proanthocyanidins, leucoanthocyanidins, and iridoids and (n-BuOH–Me_2_CO-buffer phosphate pH = 5.0 (40 : 50 : 10 v/v)) to evaluate the presence of sugars.

### 2.3. Total Polyphenol Content (TPC)

 The total polyphenol content (TPC) was determined colorimetrically by using Folin-Ciocalteu's reagent and expressed as pyrogallol, according to the Brazilian Pharmacopoeia with modifications [15]. Thus, samples of aqueous extract of *P. guineense *(1000 *μ*g/mL) were prepared in distilled water. Concentrations (10 to 30 *μ*g/mL) were measured. The standard pyrogallol (1000 *μ*g/mL) was prepared in distilled water. 80 microliters of each solution was transferred to a 25 mL flask containing distilled water (10 mL) and Folin-Ciocalteu's reagent (1 mL). The volume was completed with anhydrous sodium carbonate solution (10.75% w/v), resulting in final concentrations of 3.2 *μ*g/mL of pyrogallol. A standard curve was determined with concentrations between 1.6 and 4.8 *μ*g/mL (*r* ^2^ = 0.999). Final results were given as pyrogallol equivalents. The samples were scanned in a UV/VIS spectrophotometer (Evolution 60S, Thermo Scientific), and measurements were obtained 15 min after addition of the sodium-carbonate solution and scanning ranging from 500 to 900 nm wavelength. Distilled water was used as a blank. All measurements were performed in triplicate.

### 2.4. Bacterial Strains and Inoculum Standardization

*S. aureus* strains (*n* = 11) were isolated from clinical specimens and food. The standard strain used was *S. aureus* ATCC 25923. Strains were isolated in sheep blood agar and after identification they were stored in brain heart infusion (BHI) plus glycerol 20% v/v [16]. The *S. aureus* strains used in this study showed a resistant phenotype, by the diffusion method, to several antimicrobial agents such as beta-lactams, aminoglycosides, macrolides, fluoroquinolones, tetracycline, chloramphenicol, and lincosamides. These strains were cultured onto Mueller Hinton Agar (MHA) (Acumedia Manufacturers, Baltimore, USA) and incubated at 37°C for 18 hours. Single colonies were selected and inoculated into Mueller-Hinton broth (Acumedia Manufacturers, Baltimore, USA) to turbidity comparable to that of 0.5 McFarland standard, which is equivalent to a bacterial count of approximately 10^8^ CFU/mL.

After that, the bacterial suspension was diluted in saline (1 : 10) to obtain a final inoculum of 10^7^ CFU/mL.

### 2.5. Antimicrobial Agents

The standard reference powders of ampicillin; amoxicillin/clavulanic acid; cefoxitin; ciprofloxacin; gentamicin; meropenem were provided by Eurofarma Laboratório LTDA, Brazil. Resistance was defined for each case: ampicillin (AMP, MIC ≥ 0.25 *μ*g/mL); amoxicillin/clavulanic acid (AMC, MIC ≥ 8 *μ*g/mL); cefoxitin (CFO, MIC ≥ 4 *μ*g/mL); ciprofloxacin (CIP, MIC ≥ 4 *μ*g/mL); gentamicin (GEN, MIC ≥ 8 *μ*g/mL; meropenem (MER, MIC ≥ 16 *μ*g/mL).

### 2.6. Antimicrobial Activity

The Minimal Inhibitory Concentration (MIC) test was performed by the microdilution broth method, following the recommendations established by Clinical Laboratory Standards Institute, [17], with some modifications. Serial two-fold dilutions of aqueous extract of *P. guineense* and antimicrobial agents were prepared in sterile 96-well microplates containing Mueller Hinton broth (MHB). Five microliters of bacterial suspension were inoculated in each well to give a final concentration of 10^4^ CFU/mL. *P. guineense* extract and antimicrobial agents concentrations ranged from 7.25 to 1000 *μ*g/mL and 3.12 to 400 *μ*g/mL, respectively. The growth inhibition was demonstrated by optical density at 630 nm using a microplate reader (Thermo plate—TP Reader). Considering the total growth (100%) in the control well (MHB + bacteria), the percentage of growth reduction was attributed to the remaining wells. The MIC was reported as the lowest concentration of *P. guineense* extract or antimicrobial agents that inhibited the bacterial growth after 24 h of incubation at 37°C. In order to determine the Minimal Bactericidal Concentration (MBC), the contents of the well that showed higher or equal than 70% of growth inhibition were seeded into MHA. After 24 h of incubation at 37°C, the number of surviving *S. aureus *was determined. The MBC was defined as the lowest extract concentration at which 99.9% of the bacteria have been killed. All experiments were carried out in duplicate on two different days.

### 2.7. Determination of *In Vitro* Synergistic Activity

Combinations of *P. guineense* and antimicrobial agents were tested by the checkerboard method. The appropriate dilution of *P. guineense* extract and antimicrobial agents were performed into MHB. From these dilutions, one hundred microliters were added in 96-well microplates to obtain a final concentration equal to MIC or six dilutions lower than MIC to *P. guineense* and nine dilutions lower than MIC to antimicrobial agents. Each well received 5 *μ*L of the bacterial suspensions (10^7^ CFU/mL). Plates were incubated for 24 hours. Interpretation of the data was achieved by calculating the fractional inhibitory concentration index (FICI) as follows: (MIC of *P. guineense *in combination with antimicrobial agents/MIC of *P. guineense*)* +* (MIC of antimicrobial agents in combination with *P. guineense*/MIC of antimicrobial agents). The combination was considered to be synergistic when the FICI was ≤0.5, additive when it was 0.5 to ≤1, and antagonistic when ≥2 [18].

## 3. Results

### 3.1. Phytochemical Profile

The phytochemical profile from the *Psidium guineense *extract showed the presence of tannins, flavonoids, condensed proanthocyanidins, leucoanthocyanidins, and sugar. On the other hand, the presence of alkaloids, phenylpropanoid, and saponins was not verified. The aqueous extract of *P. guineense* yielded 3.7% (w/w) over 50 g of plant material.

Concerning the total polyphenol content, the results of this study suggest that the aqueous extract of *P. guineensis* show an important amount of such secondary metabolites. Thus, the total polyphenol content was 21.62 ± 0.40 g% (1.51%) as pyrogallol equivalent.

### 3.2. Antimicrobial Activity

MIC and MBC values of *P. guineense *extract and of the ampicillin, amoxicillin/clavulanic acid, cefoxitin, ciprofloxacin, gentamicin, and meropenem against twelve *S. aureus* strains are shown in Table 1.

The aqueous extract of *P. guineense* showed a strong activity against all *S. aureus* strains with MIC values between 250 and 500 *μ*g/mL. The *S. aureus* strains revealed a resistance profile against most antimicrobial agents tested, in particular to the beta-lactam antibiotics. The MIC values for ampicillin and cefoxitin ranged from 3.12 to 400 *μ*g/mL which showed to be less effective against the *S. aureus *strains tested. For amoxicillin/clavulanic acid, the values ranged from 3.12 to 50 *μ*g/mL. Among all *S. aureus* strains tested, seven showed to be resistant to ciprofloxacin. For meropenem, MIC values ranged from 3.12 to 25 *μ*g/mL. All strains showed to be sensitive to gentamicin, except LFBM 26, LFBM 28, LFBM 33. Four strains of *S. aureus *(LFBM 01, LFBM 26, LFBM 28, LFBM 33) showed resistance to all antimicrobial agents tested. This resistance profile selected the microorganisms for the study of the interaction between *P. guineense* extract and antimicrobial agents. The values of MBC were higher than MIC in one dilution.

### 3.3. Determination of In Vitro Synergistic Activity

The MICs obtained by the combination of the aqueous extract of *P. guineense* with ampicillin, amoxicillin/clavulanic acid, cefoxitin, ciprofloxacin, and meropenem, against *S. aureus* strains (LFBM 01, LFBM 26, LFBM 28, LFBM 33) are listed in Table 2. The minimal inhibitory concentrations of *P. guineense* extract (250 *μ*g/mL) enhanced the antistaphylococcal activity of all antimicrobial agents. The synergistic activity was detected by an eight-fold decrease in the MIC of the antimicrobial agents in the combination and determined by the FICI ≤0.5.

The combination of the aqueous extract of *P. guineense* and cefoxitin showed the lowest FICI whose values ranged from 0.125 to 0.5. The MIC of cefoxitin (individual MIC 100–400 *μ*g/mL) was lowered to 1/512 (combined MIC ranged 0.19–0.78 *μ*g/mL), when it was used in combination with the aqueous extract of *P. guineense* (MIC 250 *μ*g/mL). For *S. aureus* LFBM 26 and LFBM 33 strains, this combination was also efficient on reducing the MIC of *P. guineense* extract to 1/8 × MIC (31.25 *μ*g/mL) or 1/4 × MIC (62.25 *μ*g/mL), respectively.

The inhibition of the growth of all the *S. aureus* strains by meropenem was enhanced by *P. guineense* extract (FICI 0.5). This combination was more effective against LFBM 28 (FICI 0.25) which individual MIC of *P. guineense* (250 *μ*g/mL) lowered to 1/4 × MIC.

For *S. aureus* LFBM 33 strain, the MIC value of ciprofloxacin (individual MIC 50 *μ*g/mL) was lowered to 0.78 *μ*g/mL when combined with *P. guineense* extract (1/4 × MIC) enhancing the antistaphylococcal activity of this fluoroquinolone (FICI 0.25). All other combinations of the aqueous extract of *P. guineense* with antimicrobial agents demonstrated synergistic activity, enhancing its activity, with FICI 0.5.

## 4. Discussion

Phytochemicals have great potential as antimicrobial compounds and have been proven to have great therapeutic potential [19]. Some secondary metabolites have the ability to increase the susceptibility of the microorganism. When used in combination, these metabolites have the potential to either inhibit the modified targets or exhibit a synergy by blocking one or more of the targets in the metabolic pathway, acting as a modifier of multidrug resistance mechanisms [6].

Several studies have investigated the interactions between antibiotics and phytochemicals or crude extracts against MRSA strains [4, 7, 8, 20].

In the Myrtaceae family, there is a large variety of phytochemicals with antimicrobial activity which include terpenes present in essential oils, flavonoids [21], and tannins [22].

The genus Psidium is native to America but is distributed worldwide. Their specimens are traditionally used in the prevention or treatment of a large number of diseases. Preparations of twigs or leaves of Psidium species are extensively used for the control of gastrointestinal and respiratory disorders as well as in the treatment of skin damage [23].

Ethnopharmacological evaluation demonstrated the antimicrobial activities of *Psidium* ssp. that explain its use for the treatment of infectious diseases of the digestive, respiratory, urinary tract as well as the skin and soft tissues. Within this genus, *P. guajava* is the most studied and used species [24]. Thus, we are interested in *P. guineense*, an indigenous plant of Brazil, because it is characterized by its ample spectrum of uses including gastrointestinal disorders, infection of the genitourinary tract, treatment of colds, bronchitis, and ulcers [25].

According to Sartoratto et al. [26], strong activity is for MIC values between 50–500 *μ*g/mL, moderate activity MIC values between 600–1500 *μ*g/mL, and weak activity above 1500 *μ*g/mL. Comparing with literature results, the aqueous extract of *P. guineense* (250–500 *μ*g/mL) has a strong activity against MRSA strains tested. 

The phytochemical analysis from the leaves of *P. guineense* indicated the presence of flavonoids, hydrolysable, and condensed tannins distributed throughout the leaf tissue [27]. These results are in agreement with those obtained in the present study. Total polyphenol content 21.62 ± 0.40 g% determined in aqueous extract of *Psidium guineense* plays an important role on biological properties of this extract.

Neira González et al. [11] associated the presence of flavonoids, mainly avicularin, quercetin, and guaijaverin present in the ethanolic extract of *P. guineense*, with the activity against clinical isolated *Streptococcus mutans* strains.

 Flavonoids have the ability to complex with proteins and bacterial cells forming irreversible complexes mainly with nucleophilic amino acids. This complex often leads to inactivation of the protein and loss of its function [27].

Tannins are not crystallizable substances and when they are in the aqueous system, form colloidal solutions. The antimicrobial activity of tannins can be summarized as follows: (i) binding with proteins and adhesins, inhibiting enzymes, (ii) complexation with the cell wall and metal ions, and (iii) disruption of the plasmatic membrane [28].

Synergism of natural products and antimicrobial agents is a thrust area of phytomedicinal research, developing novel perspective of phytopharmaceuticals. The synergism of plant-derived compounds and antimicrobial agents has been evaluated previously against pathogenic microorganisms. The approach is not exclusive for extract combinations, since effective combinations between single natural products, essential oils or extracts with chemosynthetics or antibiotics have been described [6, 29].

In addition to achieving these synergistic effects, the combinations of two or more compounds are essential for the following reasons: (1) to prevent or suppress the emergence of resistant strains, (2) to decrease dose-related toxicity, as a result dosage, and (3) to attain a broad spectrum of activity [18].

In this study, a growth inhibitory effect of *P. guineense* extract on MRSA strains was observed for its combinations with beta-lactams, carbapenems, and fluoroquinolones.

The combination of *P. guineense* extract and beta-lactam may help to reduce the amount of antimicrobial agents used and deliver a medicine with similar or greater potency as antimicrobial. More importantly, since phytochemicals are structurally different from antimicrobial agents and often have different modes of action, they may provide new means of studying the mechanisms of bacterial control at a molecular level. With the increase prevalence of multidrug resistant *S. aureus*, synergism testing using various combinations of phytochemicals with antimicrobial agents could be a powerful tool in helping to select appropriate antimicrobial therapy [6, 29].

The indiscriminate use of antimicrobial agents in the treatment of bacterial infections has led to the emergence and spread of resistant strains, and it resulted in a great loss of clinical efficacy of previously effective first-line antimicrobials which results in shifting of antimicrobial treatment regimen to second-line or third-line antimicrobial agents that are often more expensive with many side effects [30]. In fact, studies have showed that crude extracts of plants possess the ability to enhance the activity of antimicrobial agents [4, 7, 8].

There is a wide list of phytochemicals which act as inhibitors, and a few of them are glycosylated flavones suppressing topoisomerase IV activity, myricetin inhibiting DnaB helicase, allicin inhibiting RNA synthesis. Corilagin, a polyphenol from *Arctostaphylos uva-ursi* is found to markedly reduce the MIC of beta-lactam agents against MRSA. According to Hemaiswarya et al. [6], there are two possibilities regarding the mechanism of action of corilagin, namely, inhibition of penicillin binding protein (PBP2a) activity or inhibiting its production.

The polyphenol epigallocatechin gallate (EGCg) from green tea is believed to be synergic with beta-lactam agents since both attacked the same target site, namely, peptidoglycan. EGCg inhibits the penicillinase produced by *S. aureus* thereby restoring the activity of penicillin. The combination of EGCg with ampicillin/sulbactam reduced the MIC_90_ to 4 *μ*g/mL from its initial value of 16 *μ*g/mL [6].

Hatano et al. [31] investigated the synergistic activity of two proanthocyanidins isolated from the fruits of the Zizyphus genus. Although the MICs of these polyphones were 512–1024 *μ*g/moL, both reduced the MIC for oxacillin to 1/2–1/16 of those in the absence of the polyphenols.

It is interesting to note that most plant secondary metabolites have weak antimicrobial activity, several orders of magnitudes less than that of common antimicrobial agents produced by bacteria and fungi. In spite of the fact that plant-derived antimicrobials are less potent, plants fight infections successfully. Hence, is becomes apparent that plants adopts a synergistic mechanism between their compounds [29].

According to Rosales-Reyes et al. [32], the use of the crude extract in our study was intentional and based on the belief that it would be the closest representation to that of traditional preparations.

Therefore, it is speculated that the efficacy of a crude extract may be due to the interplay between the different active constituents that may be present in the extract leading to better activity and/or decrease in potential toxicity of some individual constituents [33].

## 5. Conclusion

An antibacterial effect of *P. guineense* extract and a synergistic effect in combination with antimicrobial agents were reproducibly observed and might be an interesting alternative therapy for infectious diseases caused by MRSA strains. In addition, more studies, including toxicity tested *in vivo*, need to be conducted on this plant before therapeutic treatments are implemented.

## Figures and Tables

**Table 1:** MIC/MBC of the aqueous extract of *Psidium guineense* and antimicrobial agents against MRSA strains.

*Staphylococcus aureus*	MIC/MBC (*μ*g/mL)							
	AMC	AMP	CEF	CIP	GET	MER	AE	Resistance phenotype^1^
ATCC 25923	3.12/6.24	3.12/6.24	3.12/6.24	3.12/6.24	0.39/0.78	3.12/6.24	250/500	Control strain
LFBM 01	25.0/50.0	400/800	400/800	50.0/100	12.5/25.0	25.0/50.0	250/500	AMC, CFO, CIP, GET
LFBM 05	6.25/12.5	100/200	25/50.0	3.12/6.24	3.12/6.24	12.5/25.0	250/500	AMP, AZI, CFO, PEN
LFBM 08	3.12/6.24	100/200	12.5/25.0	3.12/6.24	3.12/6.24	6.25/12.5	250/500	AMP; AZI, CIP, CFO
LFBM 16	12.5/25.0	25.0/50.0	12.5/25.0	3.12/6.24	3.12/6.24	6.25/12.5	250/500	AMP, AZI, CFO, PEN
LFBM 26	50.0/100	400/800	400/800	50.0/100	25.0/50.0	25.0/50.0	250/500	AMC, AMP, CFO, CIP
LFBM 28	25.0/50.0	400/800	100/200	50.0/100	25.0/50.0	12.5/25.0	250/500	AMC, CIP, CFO, GET
LFBM 29	12.5/25.0	50.0/100	25/50.0	3.12/6.24	3.12/6.24	12.5/25.0	500/1000	AMC, AMP, CFO, PEN
LFBM 30	12.5/25.0	200/400	12.5/25.0	50.0/100	3.12/6.24	12.5/25.0	250/500	AMC, AMP, CFO, CIP
LFBM 31	12.5/25.0	100/100	12.5/25.0	50.0/100	3.12/6.24	6.25/12.5	250/500	AMP, AZI, CFO; CIP;
LFBM 32	12.5/25.0	200/200	12.5/25.0	50.0/100	3.12/6.24	12.5/25.0	250/500	AMP, CFO, CIP, PEN
LFBM 33	12.5/25.0	200/200	200/200	50.0/100	3.12/6.24	12.5/25.0	250/500	AMC, AMP, CFO, CIP

MIC: minimal inhibitory concentration; MBC: minimal bactericidal concentration; ATCC: American Type Culture Collection; LFBM: Laboratório de Fisiologia e Bioquímica de Microorganismos; AMC: amoxicillin/clavulanic acid; AMP: ampicillin; AZI: azithromycin; CFO: cefoxitin; CIP: ciprofloxacin; GET: gentamicin; PEN: penicillin; MER: meropenem; AE: aqueous extract of *P. guineense*.

^1^Resistance phenotype determined by the disk-diffusion method.

**Table 2:** Combination testing of the aqueous extract of *Psidium guineense* with antimicrobial agents against MRSA strains.

*Staphylococcus aureus*	Combination	Individual MIC (*μ*g/mL)	Combination MIC (*μ*g/mL)	Individual FIC	FIC index (FICI)	Interpretation	% MIC reduced	% reduction of viable cells^1^
LFBM 01	AE + AMC	250/25	125/0.048	0.5/0.002	0.5	Synergistic	50.0/99.8	80.21
	AE + AMP	250/400	125/0.78	0,5/0.002	0.5	Synergistic	50.0/99.8	84.43
	AE + CFO	250/400	125/0.78	0.5/0.002	0.5	Synergistic	50.0/99.8	87.04
	AE + CIP	250/50	125/0.09	0.5/0.002	0.5	Synergistic	50.0/99.8	93.49
	AE + MER	250/25	125/0.048	0.5/0.002	0.5	Synergistic	50.0/99.8	84.24

LFBM 26	AE + AMC	250/50.0	125/0.78	0.5/0.002	0.5	Synergistic	50.0/99.8	88.82
	AE + AMP	250/400	125/0.78	0.5/0.002	0.5	Synergistic	50.0/99.8	83.58
	AE + CFO	250/400	31.25/0.78	0.125/0.002	0.125	Synergistic	87.5/99.8	80.90
	AE + CIP	250/50.0	125/0.09	0.5/0.002	0.5	Synergistic	50.0/99.8	94.59
	AE + MER	250/25	125/0.048	0.5/0.002	0.5	Synergistic	50.0/99.8	80.76

LFBM 28	AE + AMC	250/12.5	125/0.024	0.5/0.002	0.5	Synergistic	50.0/99.8	83.38
	AE + AMP	250/400	125/0.78	0.5/0.002	0.5	Synergistic	50.0/99.8	89.71
	AE + CFO	250/100	125/0.19	0.5/0.002	0.5	Synergistic	50.0/99.8	86.91
	AE + CIP	250/50.0	125/0.09	0.5/0.002	0.5	Synergistic	50.0/99.8	94.45
	AE + MER	250/25.0	62.5/0.048	0.25/0.002	0.25	Synergistic	75.0/99.8	89.08

LFBM 33	AE + AMC	250/12.5	125/0.024	0.5/0.002	0.5	Synergistic	50.0/99.8	78.89
	AE + AMP	250/200	125/0.39	0.5/0.002	0.5	Synergistic	50.0/99.8	76.72
	AE + CFO	250/200	62.5/0.39	0.25/0.002	0.25	Synergistic	75.0/99.8	81.53
	AE + CIP	250/50.0	62.5/0.78	0.25/0.002	0.25	Synergistic	75.0/99.8	94.09
	AE + MER	250/12.5	125/0.024	0.5/0.002	0.5	Synergistic	50.0/99.8	81.84

MIC: minimal inhibitory concentration; FIC: fractional inhibitory concentration; AMC: amoxicillin/clavulanic acid; AMP: ampicillin; CFO: cefoxitin; CIP: ciprofloxacin; MER: meropenem; AE: aqueous extract of *P. guineense; *LFBM: Laboratório de Fisiologia e Bioquímica de Microorganismos.

%  of  MIC  reduced = (MIC_alone_ − MIC_combined_) × 100/MIC_alone_.

^1^%reduction of viable cells in wells containing the combination aqueous extract of *P. guineense* (1/2 MIC) and antimicrobials agents (1/512 MIC).
